# Prenatal opioid use as a predictor of postpartum suicide attempts among reproductive-age women enrolled in Oregon Medicaid

**DOI:** 10.1186/s12905-024-03019-w

**Published:** 2024-03-25

**Authors:** Jangho Yoon, Mandana Masoumirad, Linh N. Bui, Patrick Richard, S. Marie Harvey

**Affiliations:** 1 Division of Health Services Administration, Department of Preventive Medicine and Biostatistics, F. Edward Hebert School of Medicine, The Uniformed Services University of the Health Sciences, 4301 Jones Bridge Rd, Bethesda, MD 20814 USA; 2 Department of Health, Society, and Behavior, University of California, Irvine, CA USA; 3 Public Health Program, School of Natural Sciences, Mathematics, and Engineering, California State University, Bakersfield, CA USA; 4 College of Health, Oregon State University, Corvallis, OR USA

**Keywords:** Suicide attempts, Opioid use disorder, Pregnancy, Postpartum, Depression, Substance use

## Abstract

**Background:**

The rates of suicide and opioid use disorder (OUD) among pregnant and postpartum women continue to increase. This research characterized OUD and suicide attempts among Medicaid-enrolled perinatal women and examined prenatal OUD diagnosis as a marker for postpartum suicide attempts.

**Methods:**

Data from Oregon birth certificates, Medicaid eligibility and claims files, and hospital discharge records were linked and analyzed. The sample included Oregon Medicaid women aged 15–44 who became pregnant and gave live births between January 2008 and January 2016 (*N* = 61,481). Key measures included indicators of suicide attempts (separately for any means and opioid poisoning) and OUD diagnosis, separately assessed during pregnancy and the one-year postpartum period. Probit regression was used to examine the overall relationship between prenatal OUD diagnosis and postpartum suicide attempts. A simultaneous equations model was employed to explore the link between prenatal OUD diagnosis and postpartum suicide attempts, mediated by postpartum OUD diagnosis.

**Results:**

Thirty-three prenatal suicide attempts by any means were identified. Postpartum suicide attempts were more frequent with 58 attempts, corresponding to a rate of 94.3 attempts per 100,000. Of these attempts, 79% (46 attempts) involved opioid poisoning. A total of 1,799 unique women (4.6% of the sample) were diagnosed with OUD either during pregnancy or one-year postpartum with 53% receiving the diagnosis postpartum. Postpartum suicide attempts by opioid poisoning increased from 55.5 per 100,000 in 2009 to 105.1 per 100,000 in 2016. The rate of prenatal OUD also almost doubled over the same period. Prenatal OUD diagnosis was associated with a 0.15%-point increase in the probability of suicide attempts by opioid poisoning within the first year postpartum. This increase reflects a three-fold increase compared to the rate for women without a prenatal OUD diagnosis. A prenatal OUD diagnosis was significantly associated with an elevated risk of postpartum suicide attempts by opioid poisoning via a postpartum OUD diagnosis.

**Conclusions:**

The risk of suicide attempt by opioid poisoning is elevated for Medicaid-enrolled reproductive-age women during pregnancy and postpartum. Women diagnosed with prenatal OUD may face an increased risk of postpartum suicides attempts involving opioid poisoning.

**Supplementary Information:**

The online version contains supplementary material available at 10.1186/s12905-024-03019-w.

## Background

In 2021, 48,183 suicide deaths occurred in the U.S. (132 suicides per day) and 1.7 million suicides were attempted by adults aged 18 or older [1]. Suicide rates steadily increased during the past decades with the female suicide rate increasing 53% from 1999 to 2017, compared to a 26% increase for men [2]. Death by suicide is among the most common causes of perinatal mortality [3–8] and accounts for one-fifth of all postpartum deaths [3]. Prior studies further document that three-quarters of maternal suicides occur in the postpartum period [4, 6, 9, 10].

The literature suggests depression as a major risk factor for perinatal suicidality, with risk increasing with the severity of symptoms [11–14]. Psychiatric conditions and substance use are documented as psychiatric risk factors for postpartum suicide attempts resulting in hospitalization [15]. Some suggest that the recent increase in death by suicide is attributable partly to mortality from drug overdoses, particularly those involving opioids [16]. Opioid misuse has been linked to suicide ideation, suicide attempts, and suicide deaths [17–22]. 57% opioid overdose survivors hospitalized in a psychiatric facility reported prior suicide ideation [23].

Interestingly, opioid use and misuse have also been on the rise during the past decades, particularly among women of reproductive age. From 1999 to 2011, the age-adjusted rate of opioid poisoning deaths in the U.S. increased more than threefold [24]. The most recent estimate suggests that the number of women who received opioid-related diagnoses at the time of delivery increased by 131% from 2010 to 2017 [25]. In 2019, approximately 7% of women reported prescription opioid use during pregnancy, 1 in 5 of whom reported opioid misuse [26]. Given the increasing trends in both opioid use and suicide rates among perinatal women and a reported link between suicidal behaviors and drug-overdose mortality involving opioids, it is urgently necessary to characterize opioid use and suicidality among perinatal women and to quantify the magnitude of the association between perinatal opioid use on maternal suicidality.

This study characterized maternal opioid use disorder (OUD) and suicide attempts among Medicaid-enrolled women of reproductive age in pregnancy and one-year postpartum. The sample consisted of women of reproductive age (15–44 years) who were enrolled in Oregon Medicaid during pregnancy and postpartum between 2008 and 2016 and gave live births. Specifically, we examined whether being diagnosed with OUD during pregnancy may serve as a marker for an elevated risk of suicide attempts, particularly by opioid poisoning, among postpartum women in the first year postpartum.

## Methods

### Data and study sample

We used unique linked data from multiple independently maintained data sources in Oregon, including birth certificates, Medicaid eligibility and claims data, and hospital discharge data for women of reproductive age for years 2008–2016. The Medicaid eligibility files provided data on beneficiary enrollment and demographics (date of birth, race/ethnicity, residential ZIP codes). The Medicaid medical claims data and Oregon hospital discharge data contained information on date of service and diagnosis codes. The birth certificate data provided information on birth date and gestational age that was used to estimate the date of conception, as well as additional maternal characteristics including age, race/ethnicity, education, and marital status.

The sample included all pregnant and postpartum women of reproductive age (15–44 years) enrolled in Oregon’s Medicaid program who became pregnant and gave live births between January 1, 2008 and January 1, 2016 (*N* = 61 481). In Oregon, pregnant women with incomes up to 185% of the federal poverty level are eligible for Medicaid coverage, and Medicaid covers just under half of all births in the state [27]. We restricted the sample to include those women who were enrolled in Medicaid from the time of conception to one-year postpartum to have complete medical records on key outcome measures, such as suicide attempt and opioid use, during the entire pregnancy and also up to a year postpartum.

Ethical approval was obtained from the Institutional Review Boards at the Oregon Health Authority and Oregon State University. Stata Version 17 MP was used for statistical analysis.

### Measures

The key measures included suicide attempt and OUD, separately for the pregnancy and one-year postpartum periods. ‘Suicide attempt’ was identified using *International Classification of Diseases, Ninth Revision, Clinical Modification* (*ICD-9-CM*) and *International Classification of Diseases, Tenth Revision, Clinical Modification* (*ICD-10-CM*) diagnosis codes from Medicaid claims and hospital discharge data that occurred during pregnancy or one-year postpartum. *‘*Suicide attempt with opioid poisoning’ was defined as a subset of suicide attempts by poisoning with opioid using ICD-9/10-CM diagnosis codes from Medicaid medical claims and hospital discharge data. A detailed description of variable definitions and data sources are provided in Supplementary Material S1.

‘OUD’ was identified using ICD-9/10-CM diagnosis codes for opioid abuse and dependence retrieved from the medical claims and hospital discharges data.

Considering that the peripartum period is characterized by a high prevalence of mental illness [28, 29] and a plausible intertwined relationship exists between suicide behaviors, the use of opioid and other substances, and serious mental illness, we constructed indicators for substance use and major depression during pregnancy and postpartum, separately. ‘Substance use disorder (SUD)’ was defined as the abuse or dependence on substances other than opioids, including alcohol, cannabis, cocaine, amphetamines and other stimulants, hallucinogens, inhalants, sedatives, hypnotics, anxiolytics, psychotropics, or other/unspecified substance abuse or dependence. ‘Major depressive disorder (MDD)’ was defined as receiving at least one ICD-9/10-CM diagnosis code for major depressive disorders from the medical claims and hospital discharges data.

Maternal characteristics came from the birth certificates, Medicaid eligibility files and hospital discharge data, and included age, race/ethnicity, education, marital status, and rurality of residence. Average age of the sample was 26.0 (SD = 5.9). About 37% of the sample were married. Non-Hispanic Whites were the majority (55.3%), followed by Hispanics (30.2%). Two-thirds of the sample graduated from high school (33.0%) or had at least some college education (34.6%). Rurality was assessed using maternal residential ZIP codes and classified according to Rural Urban Commuting Area (RUCA) criteria. It was categorized as urban areas (84.2% of the sample), large rural areas (11.9%), and small and isolated rural areas (3.9%) (Supplementary Material S2).

### Statistical analysis

We summarized the measures separately for the pregnancy and one-year postpartum periods. We also summarized suicide attempts in the first year postpartum, separately for any means and opioid poisoning, based on whether a women received an OUD diagnosis during pregnancy. We used Pearson ${\chi }^{2}$ and Fisher’s exact tests to assess independence between prenatal OUD diagnosis and each of the outcome measures for postpartum suicide attempts and OUD. We used multivariate probit regression models to test whether prenatal OUD diagnosis may predict postpartum suicide attempts, controlling for prenatal SUD, prenatal MDD, and the other maternal characteristics.

To explore a hypothesized pathway through which being diagnosed with prenatal OUD may be linked to postpartum suicide attempts by opioid poisoning, we performed an explanatory analysis that examined whether a postpartum OUD diagnosis is predicted by a prenatal OUD diagnosis. Further, we hypothesized and modeled a sequential two-stage process by which a prenatal OUD diagnosis increases the risk of suicide attempts by opioid poisoning: In Stage 1, a prenatal OUD diagnosis increases the likelihood of receiving a postpartum OUD diagnosis, and in Stage 2, a postpartum OUD diagnosis, in turn, elevates the likelihood of postpartum suicide attempts by opioid poisoning. We employed a bivariate probit regression model [30, 31] to jointly estimate the two-stage binary outcomes equations. When applied properly, this approach ensures the consistency of our estimates even in the presence of potentially significant unobserved maternal characteristics that might influence both suicide attempts and prenatal OUD, such as chronic medical conditions, financial hardship, and adverse family contexts. See Supplementary Material S3 for details.

In Stage 1 of the bivariate probit model, prenatal OUD, SUD, and MDD were utilized as instrumental variables (a.k.a. exclusion restrictions). These variables are essential for consistently identifying the parameters of the simultaneous regression equations. For the bivariate probit procedure to be valid, the instrumental variables must be (a) individually and jointly significant in predicting the likelihood of receiving a postpartum OUD diagnosis in Stage 1 and (b) validly excluded from the Stage 2 regression equation. Test statistics reported at the bottom of Table 1 support the validity of the approach.

**Table 1:** Effect of prenatal OUD diagnosis on postpartum suicide attempts: bivariate probit estimates

Main explanatory variables	Suicide attempts by any means		Suicide attempts by opioid poisoning	
	Coefficients (Std. err.)	Average incremental effects^a^ (Std. err.)	Coefficients (Std. err.)	Average incremental effects^a^ (Std. err.)
***Stage 1-Auxiliary Equation (Outcome: Postpartum OUD diagnosis)***				
Prenatal OUD diagnosis	2.098^***^ (0.060)	34.28^***^ (2.15)	2.098^***^ (0.060)	34.23^***^ (2.15)
Prenatal SUD diagnosis	0.779^***^ (0.450)	4.16^***^ (0.44)	0.779^***^ (0.450)	4.17^***^ (0.44)
Prenatal MDD diagnosis	0.231^*^ (0.106)	0.77 (0.42)	0.231^*^ (0.106)	0.78 (0.42)
***Stage 2-Main Equation (Outcome: Postpartum suicide attempts)***				
Postpartum OUD diagnosis	–0.179 (0.215)	–0.027 (0.059)	0.546^**^ (0.191)	0.091^*^ (0.042)
***Validity of the instruments***				
Joint F statistic	${\chi }^{2}$=417, *p* < 0.001		${\chi }^{2}$=417, *p* < 0.001	
Exclusion restriction: LM statistic	*NR*^*2*^ = 2.428, *p* = 0.297		*NR*^*2*^ = 3.659, *p* = 0.161	

Notes: (Auxiliary) Stage 1 model specification additionally includes the maternal characteristics. (Main) Stage 2 model specification additionally includes postpartum SUD and the maternal characteristics. Full results are provided in Supplementary Material S5

^a^Measured as an average change in percentage points

^*^p < 0.05; ^*^p < 0.01; ^***^p < 0.001

We computed average incremental effects via the finite-difference method to measure changes in the outcomes in average predicted probabilities, and obtained delta-method standard errors [30, 32]. A detailed description of the multivariate models is provided in Supplementary Material S3.

## Results

**Fig. 1:**
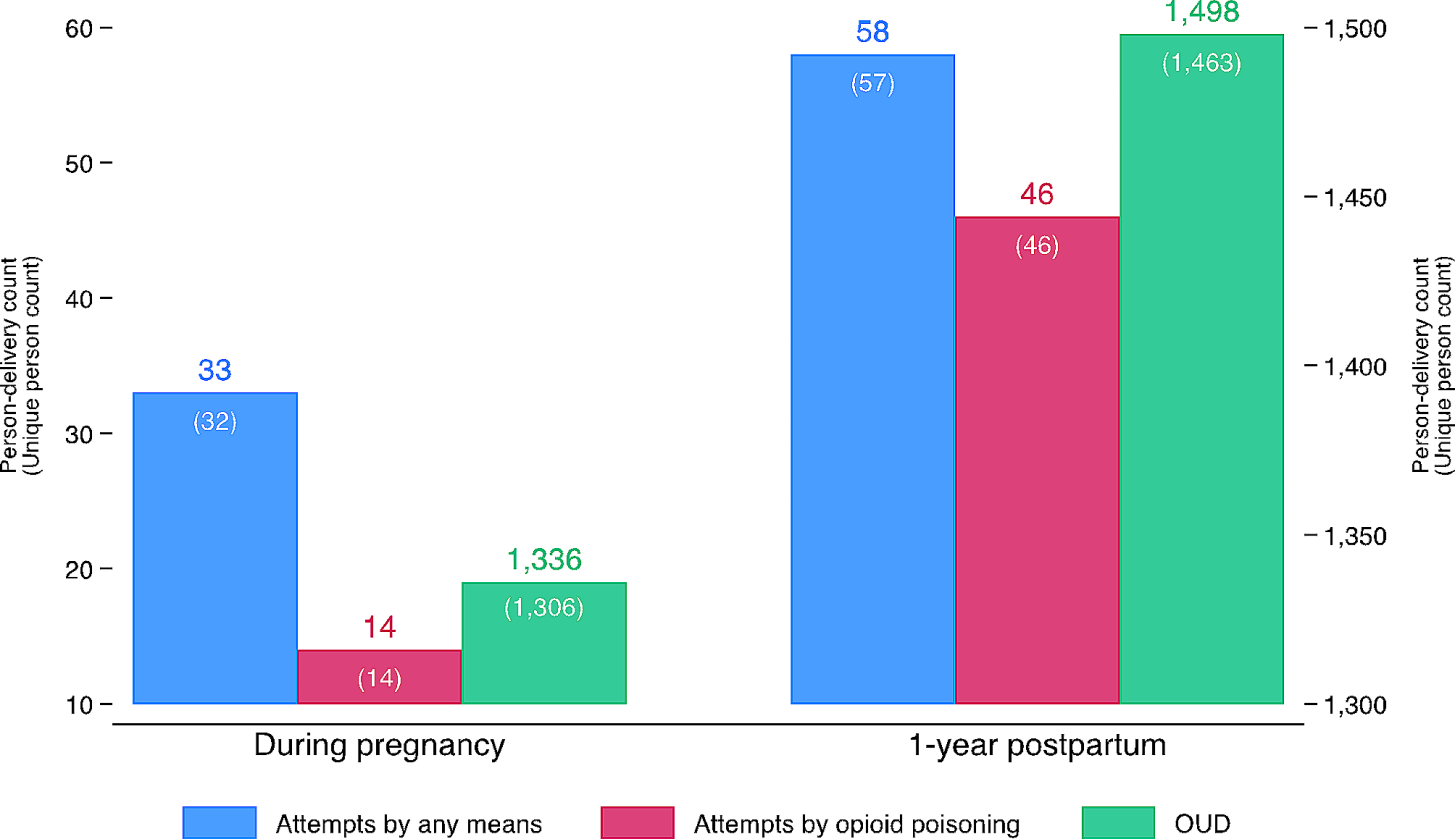
Rates of suicide attempts and OUD among Medicaid-enrolled women of reproductive age in Oregon, 2008–2016 (*N* = 61 481)

Figure 1 depicts the frequencies of suicide attempts. A total of 91 suicide attempts occurred among 88 unique women during pregnancy and in the first year postpartum, with 66% (*n* = 60 attempts) involving opioid poisoning. Suicide attempts were more frequent in the one-year postpartum period than during pregnancy. While 33 prenatal suicide attempts by any means (32 unique women) were identified, there were 58 postpartum suicide attempts by any means (57 unique women), corresponding to a rate of 94.3 attempts per 100,000. Of these postpartum suicide attempts, 79% (*n* = 46 attempts) involved opioid poisoning. The rate of suicide attempts by opioid poisoning was more than three times larger in the one-year postpartum period than during pregnancy. There were 1,799 unique women (2,834 person-deliveries or 4.6% of the entire observations) diagnosed with OUD either during pregnancy or one year postpartum. This includes 1,306 unique women (*n* = 1,336 person-deliveries) diagnosed with OUD during pregnancy and 1,463 unique persons (*n* = 1,498 person-deliveries) diagnosed with OUD in the first year postpartum. During both pregnancy and one-year postpartum period, 969 unique women received an OUD diagnosis.

**Fig. 2:**
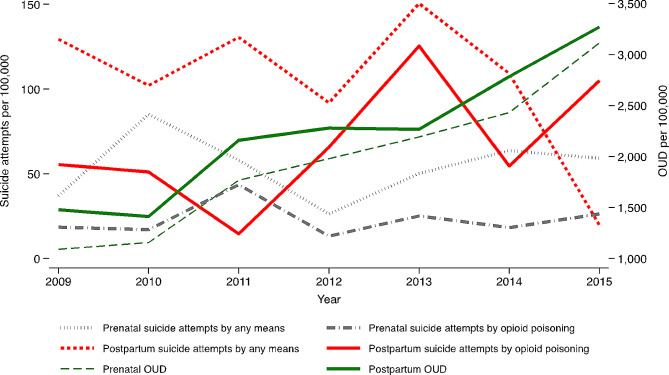
Yearly trends in the rates of suicide attempts and OUD among Medicaid-enrolled women of reproductive age in Oregon, 2009–2016

Yearly trends depicted in Fig. 2 show an overall decrease in the rate of postpartum suicide attempts by any means during the study period. In contrast, the rate of postpartum suicide attempts by opioid poisoning had an overall increase from 55.5 per 100,000 in 2009 to 105.1 per 100,000 in 2016. Suicide attempts during pregnancy overall increased slightly, for both any means and opioid poisoning. For the same period, OUD rates exhibited sharp increases. The rate of prenatal OUD diagnosis rose by 1.9 times, increasing from 1,090 per 100,000 in 2009 to 3,113 per 100,000 in 2016. Similarly, the rate of postpartum OUD increased by 1.2 times from 1,478 per 100,000 in 2009 to 3,270 per 100,000 in 2016. Taken together, the year-to-year trends imply positive relationships between prenatal OUD diagnosis, postpartum OUD diagnosis, and postpartum suicide attempts by opioid poisoning.

**Fig. 3:**
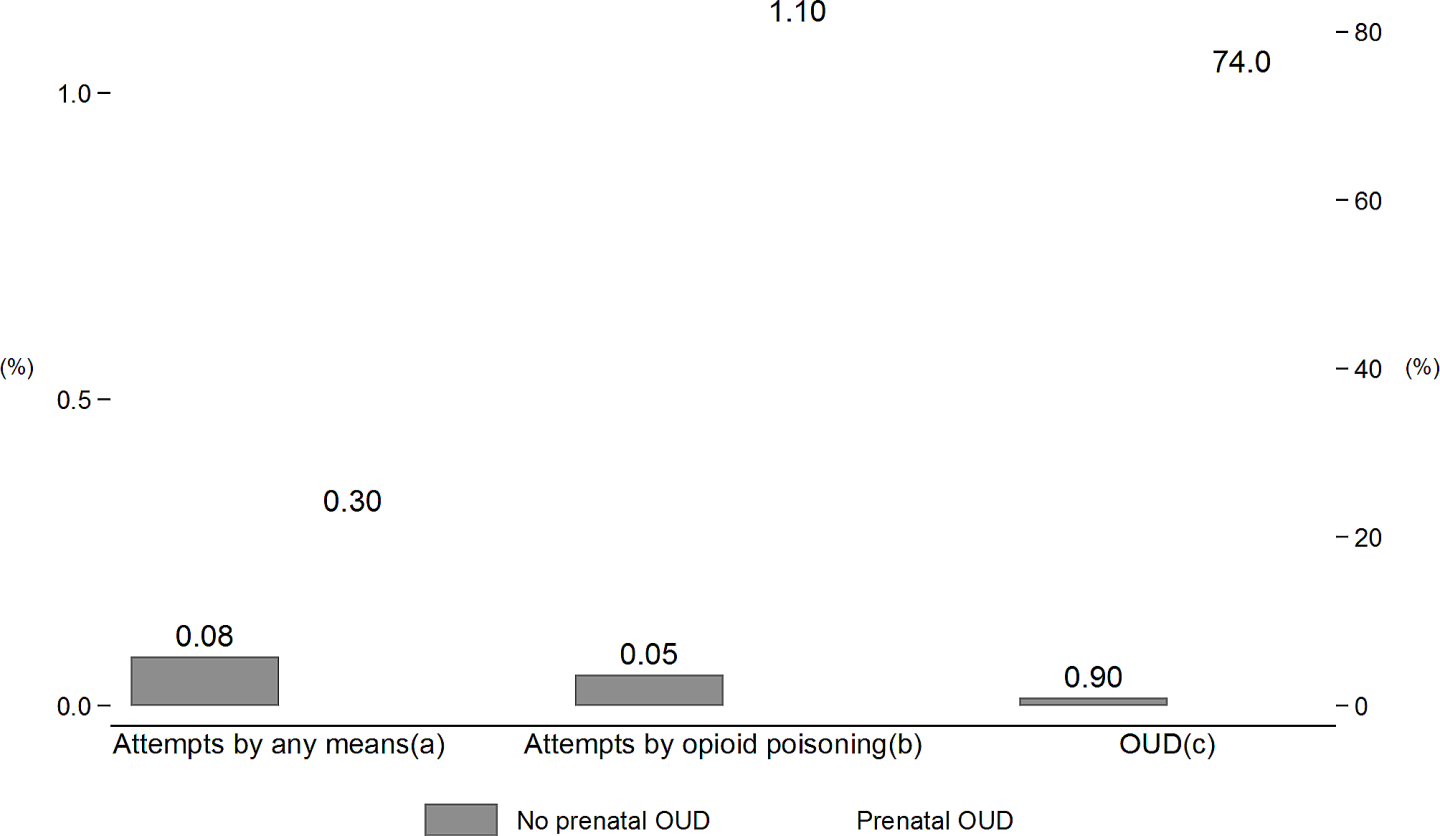
Rates of postpartum suicide attempt and OUD by the receipt of prenatal OUD diagnosis Notes: (**a**) ${\chi }^{2}$ = 7.91 (*p* = 0.024). (**b**) ${\chi }^{2}$ = 173.6 (*p* < 0.001). (**c**) ${\chi }^{2}$ = 2.9e + 04 (*p* < 0.001)

Figure 3 depicts the rates of postpartum suicide attempts and postpartum OUD diagnosis by the presence of prenatal OUD diagnosis. The rates of postpartum suicide attempts for women diagnosed with OUD during pregnancy were significantly higher than those for other women without a prenatal OUD diagnosis: 0.3% of women diagnosed with OUD during pregnancy had suicide attempts by any means during the one-year postpartum period, compared to 0.08% for those without a prenatal OUD diagnosis (${\chi }^{2}$=7.91, *p* = 0.024). A substantially larger proportion (1.1%) of women with a prenatal OUD diagnosis had postpartum suicide attempts involving opioid poisoning, compared to only 0.05% among others without a prenatal OUD diagnosis (${\chi }^{2}$=173.6, *p* < 0.001). The majority (74.0%) of women diagnosed with prenatal OUD also had a postpartum OUD diagnosis, compared to 0.9% among other women without prenatal OUD (${\chi }^{2}$=2.9e + 04, *p* < 0.001).

Estimated coefficients from the multivariate probit regression models suggest no statistically discernable association between prenatal OUD diagnosis and postpartum suicide attempts by any means (Supplementary Material S4). However, there was a positive, statistically significant association between prenatal OUD diagnosis and postpartum suicide attempt by opioid poisoning. Prenatal OUD diagnosis was also positively associated with postpartum OUD diagnosis. Results on the maternal characteristics are in line with our expectation. For example, being married was negatively associated with the likelihood of postpartum suicide attempts and OUD diagnosis. Hispanic ethnicity was negatively associated with all the postpartum outcomes. Higher education levels were negatively associated with postpartum OUD diagnosis.

**Fig. 4:**
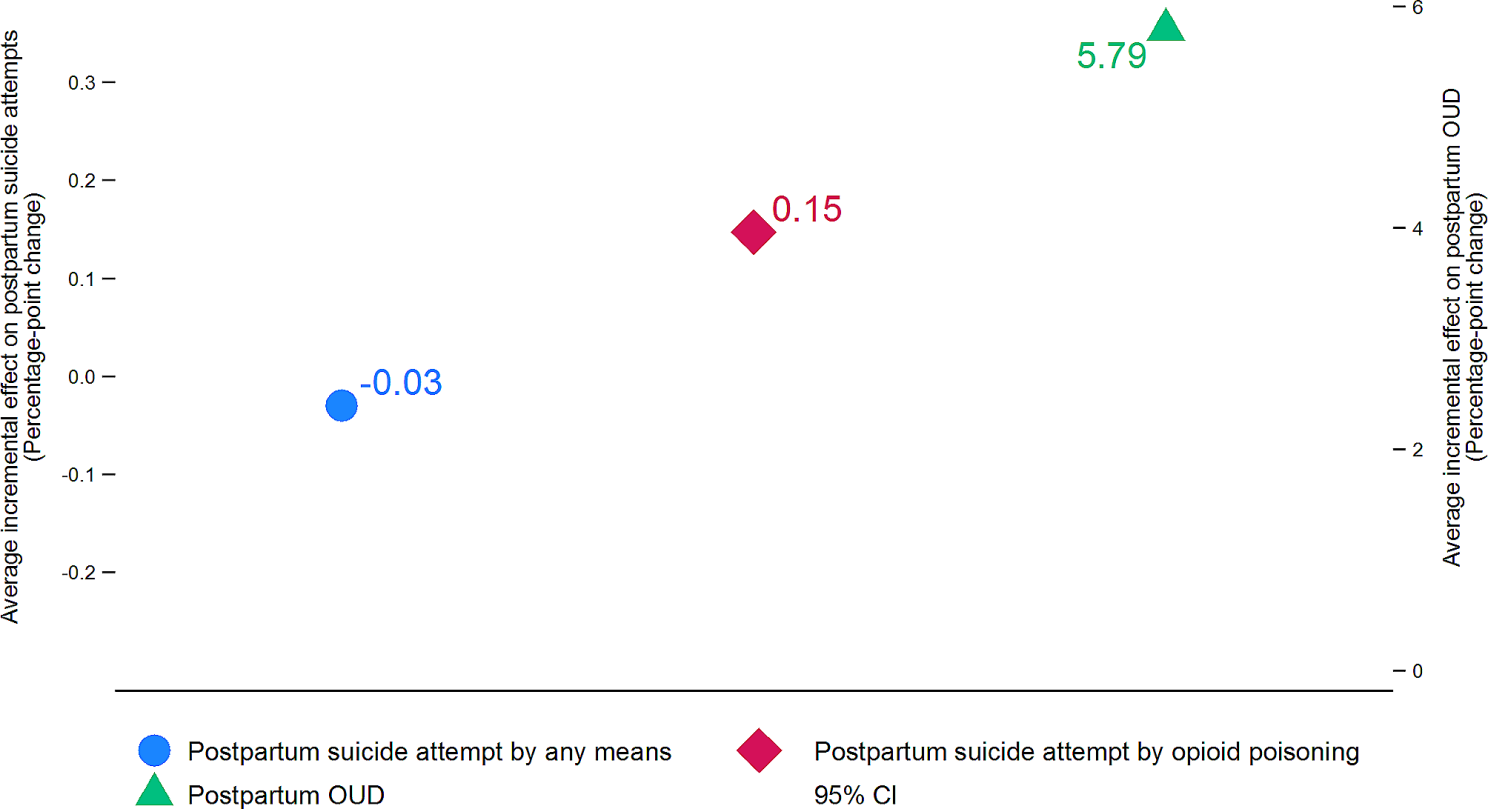
Average incremental effects of prenatal OUD diagnosis on the probabilities of postpartum suicide attempts and postpartum OUD diagnosis Notes: The point estimates and the 95% confidence intervals were calculated using the multivariate probit estimates

The average incremental effects depicted in Fig. 4 are derived from the multivariate probit estimates and measure percentage-point changes in each of the postpartum outcome measures associated with the presence of a prenatal OUD diagnosis. Prenatal OUD diagnosis on average was not significantly associated with the likelihood of postpartum suicide attempts by any means. However, it was significantly associated with a 0.15%-point increase in the likelihood of suicide attempts by opioid poisoning during the one-year postpartum period (*p* = 0.003). The discovered relationship is not trivial in magnitude; for example, the 0.15%-point increase represents a three-fold increase from the rate of postpartum suicide attempts by opioid poisoning for women who did not receive a prenatal OUD diagnosis. Receiving a prenatal OUD diagnosis was also associated with a 5.79%-point higher probability of receiving an OUD diagnosis in the first year postpartum.

Results reported in Table 1 support our hypothesized mechanism that a prenatal OUD diagnosis is linked to a postpartum OUD diagnosis and thereby elevates the risk of postpartum suicide attempts by opioid poisoning. On average, prenatal OUD diagnosis is associated with ∼ 34%-point increase in the probability of postpartum OUD diagnosis, and subsequently a postpartum OUD diagnosis is associated with a 0.09%-point increase in the probability of suicide attempts by opioid poisoning during the one-year postpartum period. Full results are provided in Supplementary Material S5.

## Discussion

The importance of perinatal mental health and suicide has garnered increasing attention in recent years. Growing evidence implies a positive link between suicide deaths and drug-overdose mortality involving opioids [16, 33–36]. Our findings indicate that many reproductive-age women enrolled in Oregon Medicaid receive diagnoses of OUD during pregnancy and postpartum and that postpartum suicide attempts occur frequently with ∼ 94 attempts per 100,000 persons. The findings align with the existing literature that highlights an elevated risk of OUD, mental illness, and deaths by suicide among peripartum women, surpassing both the general U.S. population and women of reproductive age overall [3–10, 26]. To our knowledge, this study is the first to have identified a prenatal OUD diagnosis as a significant predictor of postpartum suicide attempts. We found a positive relationship between prenatal OUD and suicide attempts by opioid poisoning among postpartum women. The discovered relationship is large in magnitude as it is three times as large as the rate of postpartum suicide attempts involving opioid poisoning among other women without a prenatal OUD diagnosis.

Our findings indicate that postpartum OUD diagnosis mediates the link between prenatal OUD diagnosis and postpartum suicide attempt, calling for close monitoring of opioid use throughout pregnancy and postpartum. We also find that prenatal SUD and MDD diagnoses significantly predict the probability of being diagnosed with OUD postpartum and suicide attempts by opioid poisoning.

This finding confirms a closely intertwined relationship between OUD, SUD, MDD, and suicidal behaviors among perinatal women as it is for the general U.S. population [33]. The literature documents a sharply elevated risk of suicide attempts among patients with psychiatric conditions who were prescribed more frequent and higher opioid doses [35], association between SUD and an increased risk of suicide [37], and prior history of MDD as a marker for suicidal ideation during pregnancy [38]. Therefore, addressing co-occurring behavioral health needs of reproductive-age women during pregnancy may further assist in identifying and mitigating the risk of opioid-involving suicidal behaviors among postpartum women.

Suicide still remains one of the most common causes of perinatal mortality, accounting for one-fifth of postpartum deaths [39]. Our findings call for policy makers and practitioners to pay close attention to the increasing trend in OUD among pregnant and postpartum women because prenatal OUD may elevate the risk of postpartum suicide behaviors. The National Action Alliance for Suicide Prevention’s Research Prioritization Task Force set forth a priority of “developing risk algorithms from health care data that can be used for suicide risk detection.” Our findings imply that similar to what was suggested by Ilgen and colleagues [34], health care providers and systems may want to consider prenatal OUD as a marker for elevated risk of suicide among postpartum women. It is also worth noting that identifying and addressing prenatal OUD may yield other positive externalities, averting various birth and neonatal complications including preterm birth, low birth weight, and neonatal abstinence syndrome. These complications are reportedly linked to maternal OUD [40, 41].

Several limitations and future directions are noteworthy. First, we were not able to examine completed suicides because only a few completed suicides were identified in our sample (*n* = 11). Notwithstanding, a prior suicide attempt is among the most important risk factor for completed suicides [42–45]. Second, we were able to utilize only 2008 through 2016 years of data. Additional, more recent data may reflect an up-to-date state of maternal OUD and suicidal behaviors. Third, we analyzed data on low-income women enrolled in Oregon Medicaid program. Generalizability may depend, therefore, on various social and system determinants of behavioral health; for example, family income and health insurance might moderate the identified link between prenatal OUD and postpartum suicidal behaviors. Fourth, we followed women up to one year postpartum. Although nine to twelve months following delivery are suggested as a critical time when postpartum women are particularly vulnerable for suicide [50], adverse effects of perinatal OUD may accumulate well beyond one year postpartum. Fifth, this study relied on ICD codes available in administrative claims data for OUD identification. Because the specificity and sensitivity of OUD ICD codes are unknown and may lead to under-identification [47], our estimates would be subject to measurement error and could be attenuated. Further, considering this limitation and recognizing the chronic nature of OUD, our findings could be more accurately interpreted as indicating an increased risk of suicide attempts by opioid poisoning, especially among those women diagnosed with OUD both during pregnancy and postpartum. Similarly, we examined suicide attempts only known to the health care system, under-identifying the true entirety of suicide attempts in the sample.

## Conclusions

Reproductive-age women enrolled in Oregon Medicaid face an elevated risk of both OUD and suicide attempts by opioid poisoning during pregnancy and postpartum, surpassing not only the general U.S. population but also women of reproductive age overall. Prenatal OUD diagnosis is a significant risk factor for postpartum suicide attempts by opioid poisoning, mediated by a postpartum OUD diagnosis.

### Electronic supplementary material

Below is the link to the electronic supplementary material.

Supplementary Material 1

## Data Availability

The datasets generated and analyzed for this study are not publicly available due to the Data Use Agreement between Oregon State University and Oregon Health Authority. Any reasonable request can be sent to the corresponding author.
