# Supplementary material for: Prenatal opioid use as a predictor of postpartum suicide attempts among reproductive-age women enrolled in Oregon Medicaid
Source: BMC Womens Health. 2024 Mar 25;24:196. doi: 10.1186/s12905-024-03019-w (PMC10964546; doi:10.1186/s12905-024-03019-w)
Supplement: Supplementary file 1 — Supplementary Material 1 [file 12905_2024_3019_MOESM1_ESM.docx]

**Supplementary Material**

**S1. Variable definitions and sources**

| **Variable** | **Definitions** | **Data sources** |
| --- | --- | --- |
| *Key measures* | | |
| Suicide attempt | = 1 if any claims coding a suicide attempt; 0 if not.  Ascertained using the following codes: ICD-9-CM codes E950-E959; ICD-10-CM codes X60-X84, Y87.0, U03. | MMC, HDD |
| Suicide attempt with opioid poisoning | = 1 if suicide attempt with opioid poisoning defined as the presence of any of medical claims coding a suicide attempt with opioid poisoning; 0 if not.  Ascertained using the following codes: Primary ICD-9-CM diagnosis codes E950-E959 with the 2^nd^-4^th^ diagnosis codes 965.00, 965.01, 965.02, 965.09, 9701; primary ICD-10-CM diagnosis codes X60-X84 with the 2^nd^-4^th^ diagnosis codes T40.0, T40.1, T40.2, T40.3, T40.4, T40.6. | MMC, HDD |
| Opioid use disorder (OUD) | = 1 if received diagnosis for opioid abuse or dependence; 0 if not.  Opioid abuse was ascertained using medical claims with one of the following diagnosis codes: ICD-9-CM code 30550-30552; ICD-10-CM code F1110, F11120-F11122, F11129, F1114, F11150, F11151, F11159, F11181, F11182, F11188, F1119. Opioid dependence was ascertained using medical claims with one of the following diagnosis codes: ICD-9-CM code 30400-30402, 30470-30472; ICD-10-CM code F1120, F11220-F11222, F11229, F1123, F1124, F11250, F11251, F11259, F11281, F11282, F11288, F1129. | MMC, HDD |
| Substance use disorder (SUD) | = 1 if received diagnosis of SUD; 0 if not.  Defined as abuse or dependence of alcohol, cannabis, cocaine, amphetamines and other stimulants, hallucinogens, inhalants, sedatives, hypnotics, anxiolytics, psychotropics, or other/unspecified substance abuse or dependence. | MMC, HDD |
| Major depressive disorder (MDD) | = 1 if received diagnosis of MDD; 0 if not.  Ascertained using ICD-9-CM codes of 296.2, 296.21-296.26, 296.3, 296.31-296.36, 300.4, 311; or ICD-10-CM codes of F32.0-F32.5, F32.9, F33, F33.1-F33.3, F33.41, F33.42, F34.1. | MMC, HDD |
| *Maternal characteristics* | | |
| Age | Age in years at the time of conception | ME, BC, HDD |
| Marital status | = 1 if married at the time of delivery; 0 if not | BC |
| Race/ethnicity | Indicators for White, Black, Asian, American Indian/Alaskan Native, Native Hawaiian/Pacific Islander, Hispanic, and Other | ME, BC |
| Education | Indicators for < high school, high school diploma, and college | BC |
| Rurality | Indicators for urban, large rural, and small/isolated rural areas | RUCA codes |

Notes: MMC=Medicaid medical claims. HDD=Hospital discharge data. ME=Medicaid eligibility files. BC=Birth certificate. RUCA=Rural-urban commuting area. Health care providers and organizations in Oregon transitioned from ICD-9-CM to ICD-10-CM on October, 1, 2015 in compliance with the rule established by the Centers for Medicare and Medicaid Services (CMS).

**S2. Sample characteristics (N=60,481)**

| **Variables** | **No, n (%)** |
| --- | --- |
| Age [mean, SD] | 26.0 (5.9) |
| *Marital status* |  |
| Not married (reference) | 37,372 (62.4) |
| Married | 23,061 (37.5) |
| Missing | 48 (0.08) |
| *Race/ethnicity* |  |
| Non-Hispanic White (reference) | 33,978 (55.3) |
| Black | 3,470 (5.7) |
| Asian | 1,443 (2.3) |
| AIAN/NHPI | 4,017 (6.5) |
| Hispanic | 18,573 (30.2) |
| Missing | 33 (0.05) |
| *Education* |  |
| < High school (reference) | 19,851 (32.5) |
| High school graduate | 20,157 (33.0) |
| Some college | 15,216 (24.9) |
| College or higher | 5,911 (9.7) |
| Missing | 346 (0.6) |
| *Rurality* |  |
| Urban (reference) | 51,415 (84.2) |
| Large rural city/town | 7,294 (11.9) |
| Small/isolated rural town | 2,380 (3.9) |

Notes: AIAN/NHPI =American Indian and Alaska Native/Native Hawaiian and Pacific Islander.

**S3. Analytic approach**

**Probit model**

The following probit regression model was estimated to identify the ‘total’ effect of receiving diagnosis of opioid use disorder (OUD) in pregnancy on the probability of suicide attempts and OUD, diagnosis in the first year postpartum:

(1) $Pr(pp\_y=1)=\Phi\left( \alpha\cdot pn\_oud+x'\cdot\beta\right)$

Here $\Phi$ is the cumulative distribution function of the standard normal distribution. $pp\_y$ is one of the binary postpartum outcomes (suicide attempt by any means, suicide attempt by opioid poisoning, and OUD diagnosis). The main explanatory variable $pn\_oud$ indicates any OUD diagnosis during pregnancy and therefore its coefficient $\alpha$ captures the overall relationship between prenatal OUD and the probability of postpartum suicide attempt, for example. Covariates specified in $x'$ included prenatal substance use disorder (SUD) diagnosis, prenatal major depressive disorder (MDD) diagnosis to adjust for the interwoven relationships between OUD, SUD, and MDD, as well as the maternal characteristics (age, race/ethnicity, education, marital status, and rurality/urbanity of residence).

**Simultaneous equations and bivariate probit model**

We hypothesized and explored a pathway through which prenatal OUD diagnosis affects postpartum suicide attempt using a system of two sequential structural equations as following:

(2) Stage 1: $Pr(pp\_oud=1)=\Phi\left( \gamma^{1}pn\_oud+ z1'\cdot\delta^{1}+ x'\cdot\beta^{1}+\epsilon^{1} \right)$

(3) Stage 2: $Pr(pp\_att=1)=\Phi\left( \gamma^{2}\cdot pp\_oud+ pp\_sud\cdot\delta^{2}+ x'\cdot\beta^{2}+\epsilon^{2} \right)$

Here $pp\_oud$ and $pp\_att$ are binary variables indicating postpartum OUD diagnosis and postpartum suicide attempt, respectively. We hypothesized that prenatal OUD diagnosis increases the risk of postpartum OUD diagnosis and thereby postpartum suicide attempt. Therefore, the coefficients on the $pn\_oud$ and $pp\_oud$ variables are of main interest: $\gamma^{1}$ measures the effect of prenatal OUD diagnosis on postpartum OUD diagnosis, and $\gamma^{2}$ captures the effect of postpartum OUD diagnosis on postpartum suicide attempt. Taken together, the main coefficients serve as a test of whether prenatal OUD diagnosis can predict postpartum OUD diagnosis and subsequently postpartum suicide attempt. We specified prenatal SUD diagnosis and prenatal MDD diagnosis in the vector $z1'$ in Stage 1 and postpartum SUD diagnosis ($pp\_sud$) in Stage 2, so that the additional variables could serve as exclusion restrictions when the equations were estimated jointly.

We estimated the system of simultaneous equations jointly using the bivariate probit procedure, maintaining its standard assumptions that the errors ($\epsilon^{1}$ and $\epsilon^{2}$) are normally distributed with means of ‘0’ and standard deviations of ‘1’ (33, 34). In so doing, we strived to obtain consistent estimates when both outcomes are dichotomous. This procedure recognizes that certain unobserved person characteristics related to postpartum suicide attempt and prenatal OUD diagnosis may be linked (e.g., underlying medical conditions or financial hardship associated with suicide behaviors and opioid use) but would yield consistent estimates if the equations with binary outcome variables are estimated jointly via the bivariate probit procedure.

**Average incremental effects**

Estimated probit and bivariate probit coefficients are not directly interpretable in terms of predicted probabilities; instead, the coefficients can be interpreted only in terms of direction of a relationship and its statistical significance (35). Therefore, we additionally calculated average incremental effects ($\pi$) using estimated coefficients. We applied the finite-difference method, which measures the average impact of a discrete change in a discrete variable on the probability of the outcomes (35). For example, in Equation 1, we calculated an average difference in the probability of postpartum suicide attempt ($pp\_att$) between individuals with prenatal OUD diagnosis ($pn\_oud=1$) and others without prenatal OUD ($pn\_oud=0$):

(4) $\pi=\frac{\sum\left( \Pr(pp\_att=1)|pn\_oud=1 \right)-\left( \Pr(pp\_att=1)|pn\_oud=0 \right)}{N}$

Standard errors were obtained using the delta method (35).

**S4. Total effects of prenatal OUD on postpartum suicide attempt and postpartum OUD: probit regression coefficients**

| **Variables** | **Postpartum outcomes** | | |
| --- | --- | --- | --- |
|  | **Suicide attempt by any means** | **Suicide attempt by opioid poisoning** | **OUD** |
| Prenatal OUD | –0.0964 | 0.6118^**^ | 2.0977^***^ |
|  | (0.2002) | (0.1899) | (0.0599) |
| Prenatal SUD | 0.5649^***^ | 0.3171^*^ | 0.7792^***^ |
|  | (0.1222) | (0.1607) | (0.0496) |
| Prenatal MDD | 0.4248 | n/a^a^ | 0.2314^*^ |
|  | (0.2188) |  | (0.1056) |
| Age | –0.0171 | 0.0035 | 0.0123^***^ |
|  | (0.0090) | (0.0078) | (0.0027) |
| Married | –0.2625^*^ | –0.1208 | –0.2330^***^ |
|  | (0.1086) | (0.1070) | (0.0366) |
| *Race/ethnicity (reference: White)* | | | |
| Black | 0.1652 | –0.0204 | –0.3642^***^ |
|  | (0.1416) | (0.1866) | (0.0831) |
| Asian | n/a^a^ | 0.0281 | –0.1594 |
|  |  | (0.3010) | (0.1253) |
| AIAN/NHPI | 0.1791 | –0.0255 | –0.0649 |
|  | (0.1279) | (0.1624) | (0.0604) |
| Hispanic | –0.3095^*^ | –0.2826^*^ | –0.4009^***^ |
|  | (0.1208) | (0.1388) | (0.0451) |
| *Education (reference: Less than high school)* | | | |
| High school graduate | –0.0316 | 0.0414 | 0.0139 |
|  | (0.0987) | (0.1143) | (0.0403) |
| Some college | –0.1212 | –0.0125 | –0.0041 |
|  | (0.1173) | (0.1236) | (0.0432) |
| College or higher | –0.0615 | –0.5047 | –0.3248^***^ |
|  | (0.1898) | (0.2855) | (0.0716) |
| *Rurality (reference: Urban)* | | | |
| Large rural city/town | 0.0773 | –0.2987 | –0.1208^*^ |
|  | (0.1134) | (0.2059) | (0.0546) |
| Small/isolated rural town | –0.1141 | 0.1525 | –0.0505 |
|  | (0.2087) | (0.1866) | (0.0828) |
| *Year of delivery (reference: 2008)* | | | |
| 2009 | –0.0513 | –0.3177 | –0.0196 |
|  | (0.2549) | (0.2754) | (0.1260) |
| 2010 | –0.1239 | –0.3121 | –0.0852 |
|  | (0.2551) | (0.2785) | (0.1264) |
| 2011 | –0.1042 | –0.7244^*^ | 0.0598 |
|  | (0.2465) | (0.3546) | (0.1218) |
| 2012 | –0.1510 | –0.2991 | 0.0084 |
|  | (0.2542) | (0.2551) | (0.1205) |
| 2013 | –0.0156 | –0.1608 | –0.0577 |
|  | (0.2427) | (0.2410) | (0.1213) |
| 2014 | –0.0924 | –0.3744 | 0.0894 |
|  | (0.2433) | (0.2550) | (0.1180) |
| 2015 | –0.6019^*^ | –0.1752 | 0.0706 |
|  | (0.2790) | (0.2374) | (0.1168) |
| Intercept | –2.4703^***^ | –2.9655^***^ | –2.6008^***^ |
|  | (0.2839) | (0.3007) | (0.1293) |
|  |  |  |  |
| *N* | 59,092 | 59,811 | 60,501 |
| Log-likelihood | –415.0 | –331.7 | –3366.8 |

Notes: OUD=Opioid use disorder. SUD=Substance use disorder. MDD=Major depressive disorder. Reported in parentheses are robust standard errors. All models are adjusted for age, marital status, race/ethnicity, and rurality of residence.

^a^Excluded because of perfect prediction.

^*^p<0.05. ^**^p<0.01. ^***^p<0.001.

**S5. Effect of prenatal OUD on postpartum suicide attempt: bivariate probit coefficients**

| **Variables** | **Suicide attempt by any means** | **Suicide attempt by opioid poisoning** |
| --- | --- | --- |
| ***Stage 1-Auxiliary Equation (Outcome: Postpartum OUD)*** | | |
| Prenatal OUD | 2.0983^***^ | 2.0970^***^ |
|  | (0.0599) | (0.0599) |
| Prenatal SUD | 0.7786^***^ | 0.7796^***^ |
|  | (0.0496) | (0.0496) |
| Prenatal MDD | 0.2299^*^ | 0.2337^*^ |
|  | (0.1054) | (0.1056) |
| Age | 0.0123^***^ | 0.0123^***^ |
|  | (0.0027) | (0.0027) |
| Married | –0.2330^***^ | –0.2336^***^ |
|  | (0.0366) | (0.0366) |
| *Race/ethnicity (reference: White)* | | |
| Black | –0.3638^***^ | –0.3656^***^ |
|  | (0.0831) | (0.0831) |
| Asian | –0.1595 | –0.1578 |
|  | (0.1253) | (0.1252) |
| AIAN/NHPI | –0.0653 | –0.0650 |
|  | (0.0604) | (0.0604) |
| Hispanic | –0.4012^***^ | –0.4012^***^ |
|  | (0.0451) | (0.0451) |
| *Education (reference: Less than high school)* | | |
| High school graduate | 0.0136 | 0.0132 |
|  | (0.0403) | (0.0403) |
| Some college | –0.0042 | –0.0040 |
|  | (0.0432) | (0.0432) |
| College or higher | –0.3250^***^ | –0.3251^***^ |
|  | (0.0716) | (0.0717) |
| *Rurality (reference: Urban)* |  |  |
| Large rural city/town | –0.1206^*^ | –0.1217^*^ |
|  | (0.0546) | (0.0546) |
| Small/isolated rural town | –0.0509 | –0.0486 |
|  | (0.0829) | (0.0827) |
| *Year of delivery (reference: 2008)* | | |
| 2009 | –0.0182 | –0.0147 |
|  | (0.1259) | (0.1259) |
| 2010 | –0.0833 | –0.0802 |
|  | (0.1262) | (0.1263) |
| 2011 | 0.0610 | 0.0650 |
|  | (0.1216) | (0.1216) |
| 2012 | 0.0101 | 0.0138 |
|  | (0.1203) | (0.1203) |
| 2013 | –0.0558 | –0.0528 |
|  | (0.1210) | (0.1211) |
| 2014 | 0.0909 | 0.0944 |
|  | (0.1179) | (0.1179) |
| 2015 | 0.0722 | 0.0752 |
|  | (0.1166) | (0.1166) |
| Intercept | –2.6014^***^ | –2.6045^***^ |
|  | (0.1292) | (0.1292) |
| ***Stage 2-Main Equation (Outcome: Postpartum suicide attempt)*** | | |
| Postpartum OUD | –0.0978 | 0.4823^*^ |
|  | (0.2135) | (0.1966) |
| Postpartum SUD | 0.9158^***^ | 0.7127^***^ |
|  | (0.1038) | (0.1512) |
| Age | –0.0236^*^ | –0.0022 |
|  | (0.0099) | (0.0094) |
| Married | –0.2236^*^ | –0.0493 |
|  | (0.1133) | (0.1179) |
| *Race/ethnicity (reference: White)* | | |
| Black | 0.2244 | 0.0266 |
|  | (0.1483) | (0.2017) |
| Asian | –3.3084^***^ | 0.1478 |
|  | (0.0940) | (0.3292) |
| AIAN/NHPI | 0.1875 | –0.0348 |
|  | (0.1348) | (0.1760) |
| Hispanic | –0.2222 | –0.1953 |
|  | (0.1237) | (0.1479) |
| *Education (reference: Less than high school)* | | |
| High school graduate | –0.0003 | 0.0316 |
|  | (0.1004) | (0.1217) |
| Some college | –0.0765 | 0.0101 |
|  | (0.1229) | (0.1329) |
| College or higher | 0.0562 | –0.4246 |
|  | (0.1976) | (0.3075) |
| *Rurality (reference: Urban)* |  |  |
| Large rural city/town | 0.1019 | –0.4042 |
|  | (0.1199) | (0.2069) |
| Small/isolated rural town | –0.0871 | 0.2312 |
|  | (0.2231) | (0.2024) |
| *Year of delivery (reference: 2008)* | | |
| 2009 | 0.0040 | –0.2699 |
|  | (0.2552) | (0.2836) |
| 2010 | –0.0964 | –0.2637 |
|  | (0.2551) | (0.2873) |
| 2011 | –0.0668 | –0.6895 |
|  | (0.2470) | (0.3899) |
| 2012 | –0.1612 | –0.2373 |
|  | (0.2537) | (0.2613) |
| 2013 | 0.0035 | –0.0912 |
|  | (0.2420) | (0.2453) |
| 2014 | –0.0741 | –0.3402 |
|  | (0.2448) | (0.2639) |
| 2015 | –0.6043^*^ | –0.1422 |
|  | (0.2842) | (0.2412) |
| Intercept | –2.5222^***^ | –3.1161^***^ |
|  | (0.2994) | (0.3340) |
|  |  |  |
| *N* | 60,501 | 60,501 |
| Log-likelihood | –3752.5 | –3654.4 |

Notes: OUD=Opioid use disorder. SUD=Substance use disorder. MDD=Major depressive disorder. Reported in parentheses are robust standard errors.

^*^p<0.05. ^**^p<0.01. ^***^p<0.001.
